# The Cell-Autonomous Pro-Metastatic Activities of PD-L1 in Breast Cancer Are Regulated by N-Linked Glycosylation-Dependent Activation of STAT3 and STAT1

**DOI:** 10.3390/cells12192338

**Published:** 2023-09-23

**Authors:** Nofar Erlichman, Tsipi Meshel, Tamir Baram, Alaa Abu Raiya, Tamar Horvitz, Hagar Ben-Yaakov, Adit Ben-Baruch

**Affiliations:** The Shmunis School of Biomedicine and Cancer Research, George S. Wise Faculty of Life Sciences, Tel Aviv University, Tel Aviv 6997801, Israel; nofarerlichman@gmail.com (N.E.); tsipi.meshel@gmail.com (T.M.); tamiros21@gmail.com (T.B.); alaaaboraiya1@gmail.com (A.A.R.); tamarhorvits@gmail.com (T.H.); etzion.hagar1@gmail.com (H.B.-Y.)

**Keywords:** Breast cancer, N-linked glycosylation, PD-1, PD-L1, STAT1, STAT3

## Abstract

PD-L1 has been characterized as an inhibitory immune checkpoint, leading to the suppression of potential anti-tumor immune activities in many cancer types. In view of the relatively limited efficacy of immune checkpoint blockades against PD-L1 in breast cancer, our recent study addressed the possibility that in addition to its immune-inhibitory functions, PD-L1 promotes the pro-metastatic potential of the cancer cells themselves. Indeed, our published findings demonstrated that PD-L1 promoted pro-metastatic functions of breast cancer cells in a cell-autonomous manner, both in vitro and in vivo. These functions fully depended on the integrity of the S283 intracellular residue of PD-L1. Here, using siRNAs and the S283A-PD-L1 variant, we demonstrate that the cell-autonomous pro-metastatic functions of PD-L1—tumor cell proliferation and invasion, and release of the pro-metastatic chemokine CXCL8—required the activation of STAT3 and STAT1 in luminal A and triple-negative breast cancer cells. The cell-autonomous pro-metastatic functions of PD-L1 were potently impaired upon inhibition of N-linked glycosylation (kifunensine). Site-specific mutants at each of the N-linked glycosylation sites of PD-L1 (N35, N192, N200, and N219) revealed that they were all required for PD-L1-induced pro-metastatic functions to occur; the N219 site was the main regulator of STAT3 and STAT1 activation, with accompanying roles for N192 and N200 (depending on the cell type). Using a T cell-independent mouse system, we found that cells expressing N35A-PD-L1 and N219A-PD-L1 had a significantly lower tumorigenic and metastatic potential than cells expressing WT-PD-L1. TCGA analyses revealed significant associations between reduced survival and high levels of α-mannosidase II (inferring on N-linked glycosylation) in breast cancer patients. These findings suggest that N-linked glycosylation of PD-L1 may be used to screen for patients who are at greater risk of disease progression, and that modalities targeting N-linked glycosylated PD-L1 may lead to the inhibition of its cell-autonomous pro-metastatic functions and to lower tumor progression in breast cancer.

## 1. Introduction

Immunotherapies, whose aim is to potentiate anti-tumor immune activities, are now being implemented in the treatment of melanoma and of tumors with high mutation rates and DNA mismatch repair defects [1,2,3]. In breast cancer, immune checkpoint blockades (ICBs) have been introduced to the treatment of triple-negative breast cancers (TNBCs) that demonstrate relatively high genomic instability [4,5]. TNBC is a most aggressive subtype of breast cancer, one of the four major breast cancer subtypes that also include the prevalent luminal A subtype, and the luminal B and HER2+ subtypes [6].

Clinical studies in the last several years have indicated that the response rates to ICBs in TNBC patients are limited [4,5]. These observations emphasize the need to better identify the mechanisms leading to insufficient success rates of ICBs, and to better understand their potential use in other breast cancer subtypes. In this context, we have recently addressed the possibility that PD-L1 carry out broader pro-metastatic activities than immune suppression, and explored the ability of PD-L1 to induce cell-autonomous pro-metastatic phenotypes and functions in TNBC and luminal A breast cancer cells. The relevance of this research direction was reinforced by the fact that PD-L1 is expressed by the tumor cells in patient biopsies in both TNBC and luminal A subtypes, with a more prevalent expression in the first than in the latter [7,8,9].

Our published study demonstrated that by being expressed in TNBC and luminal A breast cancer cells, PD-L1 has acted in a cell-autonomous manner to elevate the pro-metastatic activities of the tumor cells [10]. PD-L1 has promoted the growth of the cells, their ability to release the pro-metastatic chemokine CXCL8 and to invade; all these functions were elevated by exposure to PD-1 in vitro, and have led to increased tumor growth and metastasis in a T cell-deficient mouse model system [10]. Taken together, these findings revealed pro-metastatic roles for PD-L1 that were not connected to its immune-suppressive functions, but rather to cell-autonomous activities exerted in the cancer cells themselves, leading to increased disease progression.

In additional analyses, we have demonstrated that all PD-L1 activities in the tumor cells depended on the integrity of the S283 intracellular residue of PD-L1 [10]. The cardinal roles played by S283 were revealed at multiple tumor-promoting levels, including tumor cell proliferation, CXCL8 release and invasion, as well as in vivo [10]. In the T cell-deficient animal model system, cells expressing the S283-mutated PD-L1 variant demonstrated poor tumorigenic and metastatic phenotypes [10].

In the current research, we have taken a step further in deciphering the processes mediating the pro-metastatic roles of PD-L1 in breast tumor cells, at the cell-autonomous and PD-1-induced levels. PD-L1 lacks canonical protein–protein interaction motifs, thus presenting a challenge in identifying its mechanisms of action. Recent studies proposed that the mTOR/AKT pathways are the ones mainly mediating the intrinsic activities of PD-L1 in several cancer systems [11,12,13,14]. The interactions of the mTOR cascade with STAT-mediated signaling (e.g., in immunity [15]), and the roles attributed to STAT3 and STAT1 in promoting PD-L1 expression [16,17,18,19], have led us to inquire in depth about the potential involvement of STAT3 and STAT1 in PD-L1-mediated effects in breast cancer cells. This research direction was further enforced by studies demonstrating that STAT3 and STAT1 have cardinal roles in regulating cancer progression, including in breast cancer [18,20].

Here, we demonstrate that the ability of PD-L1 to induce cell-autonomous- and PD-1-induced effects is mediated through STAT3 and STAT1 activation, leading to increased tumor cell growth, CXCL8 release and cancer cell invasion in luminal A and TNBC breast cancer cells. Moreover, we have extended the search for mechanisms that control PD-L1 functions and asked whether PD-L1 needs to be N-linked glycosylated in order to exert its cell-autonomous, tumor-promoting functions in these cells.

Previous studies have demonstrated that N-linked glycosylation of PD-L1 increased PD-1 binding, leading to more efficient inhibition of anti-tumor T cell activities; it was also found to interfere with the binding of antibodies used for PD-L1 diagnosis and PD-L1-directed therapy [21,22,23,24,25,26]. PD-L1 N-linked glycosylation was noted in breast cancer patient tumors, and following removal of N-linked glycosylation, PD-L1 levels correlated with response rates to anti-PD-L1 therapy [23,24].

Adding to these findings, we now demonstrate that PD-L1 has to be glycosylated at each of its N-linked glycosylation sites (N35, N192, N200 and N219; [25,26,27]) in order to exert cell-autonomous tumor-promoting functions in breast tumor cells in vitro. In this context, we have also identified the N-linked glycosylation sites that are necessary for STAT3 and STAT1 activation. Furthermore, our findings indicate that specific PD-L1 N-linked glycosylation sites are required for its ability to promote tumor growth and metastasis in vivo, and shed light on the clinical implications of these findings in breast cancer patients (using the TCGA dataset).

Overall, in this study we demonstrate that the cell-autonomous pro-metastatic functions of PD-L1 in breast cancer cells are fully dependent on all four N-linked glycosylation sites of the protein, and that N-linked glycosylation of PD-L1 at specific sites leads to STAT3 and STAT1 activation. Consequently, PD-L1 promotes breast tumor cell growth, CXCL8 release and invasion, as well as tumor growth and metastasis in vivo. These PD-L1-induced processes have clinical implications, suggesting that inhibitory measures directed at N-linked glycosylation may apply in breast cancer therapy.

## 2. Materials and Methods

### 2.1. Cell Growth

Human MCF-7 Luminal A cells and human MDA-MB-231 (MDA) TNBC cells (both from ATCC) were grown in DMEM medium. Medium was supplemented with 10% fetal bovine serum (FBS), 2% L-glutamine and 1% penicillin-streptomycin-amphotericin solution (from Biological Industries, Beit Ha’emek, Israel and Sigma-Aldrich, St. Louis, MO, USA).

To determine the effects of PD-1 stimulation on the cells, a recombinant PD-1-IgG1 Fc chimera protein (endotoxin free; #1086-PD; R&D Systems, Minneapolis, MN, USA) was used at 2 μg/mL for 72 h. Recombinant human IgG1-Fc (#110-HG; R&D Systems) was used as control at similar conditions. Concentrations were selected based on preliminary experiments performed beforehand (as mentioned in [10]).

When relevant, MCF-7 and MDA cells were treated with kifunensine (50 μM; #K1140, Sigma-Aldrich) and/or swainsonine (50 μM; #S8195, Sigma-Aldrich) dissolved in double-distilled water. Following kinetics studies (see figures), analyses were performed of MCF-7 cells and MDA cells exposed to kifunensine or its vehicle for 48 and 72 h, respectively.

### 2.2. Western Blot

Cells were lysed in RIPA buffer, and Western blot (WB) analyses were performed using antibodies from Cell Signaling Technology (CST, Danvers, MA, USA), unless otherwise indicated: total (T)-STAT3: #4904; Phosphorylated (P)-STAT3-Y705: #9145; (T)-STAT1: #9172; (P)-STAT1: #9167; PD-L1: (#51296S). Antibodies to Calnexin (#2679S; Abcam, Cambridge, UK) or GAPDH (#ab9485; Abcam) served as loading controls. The membranes were incubated with streptavidin-horseradish peroxidase (HRP)-conjugated goat anti-rabbit IgG (#111-035-003; Jackson ImmunoResearch Laboratories, West Grove, PA, USA). The membranes were subjected to enhanced chemiluminescence (ECL) analysis (#WBLUR0500, Merck, Darmstadt, Germany), and were visualized with Amersham ImageQuant 800 (GE Healthcare, Little Chalfont, UK).

Densitometry graphs of STAT3 and STAT1 activation were generated with the ImageJ software (Version 1.53t); they present arbitrary units of pSTAT3/GAPDH or pSTAT1/GAPDH values in cells expressing WT-PD-L1 or different PD-L1 N-linked glycosylation mutants. % inhibition of STAT3 and STAT1 activation in PD-L1 mutants compared to WT-PD-L1 were calculated as “1 minus [the value of STAT3 or STAT1 activation in a PD-L1 mutant, divided by the value of STAT3 or STAT1 activation in WT-PD-L1]”.

### 2.3. STAT3 and STAT1 Down-Regulation by siRNA

The expression of STAT3 and STAT1 in MCF-7 cells and MDA cells was knocked down (KD) by transient siRNA transfection (10nM; Based on titration analyses), using Lipofectamine RNAiMAX transient reagent (#13778075; Invitrogen, Grand Island, NY, USA) according to manufacturer’s instructions, in a reverse transfection protocol. The following ON-TARGET plus siRNA SMART pools were used (all from Dharmacon, Lafayette, CO, USA): STAT3 siRNA (siSTAT3): #L-002000-00; STAT1 siRNA (siSTAT1): #L-003543-00. siRNA control (siCTRL) was introduced by ON-TARGET plus non-targeting control siRNA pool (#D-001810-10). At 72 h after transfection, cells were used in assays, as necessary. KD of STAT3 and STAT1 expression was validated by WB analyses.

### 2.4. Flow Cytometry Analyses of PD-L1 Expression

Cell surface expression of PD-L1 was determined by flow cytometry using mouse IgG1 antibodies against human PD-L1 (#14-5983-82, Thermo Fisher Scientific, Waltham, MA, USA), followed by FITC-conjugated goat anti-mouse IgG antibodies (#115-095-003, Jackson ImmunoResearch Laboratories). Baseline staining was determined by isotype-matched control mouse IgG antibodies (#400102; Biolegend, San Diego, CA, USA). Fluorescence was determined by Flow Cytometer S100EXi (Stratedigm, San Jose, CA, USA), using CELLCAPTURE software (Version 1; Stratedigm), and analyzed by FLOWJO V10 (Version 10.7.1; BD Biosciences, Franklin Lakes, NJ, USA).

### 2.5. Cancer Cell Growth In Vitro

Analyses of cell growth rates were conducted by plating the cells of different treatments at equal numbers. Cell numbers were determined after 72 h in culture using trypan blue exclusion assay in ≥2 replicates/cell type. When relevant, glycosylation inhibitors or their vehicle control were added to cell cultures one day after cell culturing, and cell counts were performed 72 h later.

### 2.6. Invasion

Cancer cells were plated in transwells with 8 μm-pore membranes (#3422, Sigma-Aldrich) coated by 20 μg/mL matrigel (#7058006, Sigma-Aldrich). The bottom wells were filled with medium that was supplemented with 10% FBS. Invasion of MCF-7 cells and MDA cells was determined after 21.5 h or 11 h of migration, respectively.

In specific experiments, the cells were stimulated with PD-1 or its control (as above); In other assays, the cells were treated with kifunensine or its vehicle (as above); in cases in which both PD-1 and kifunensine were used, the cells were first treated by kifunensine or its vehicle (as above) for 48 h for MCF-7 cells or 72 h for MDA cells. Then, the cells were stimulated with PD-1 or its control (as above) for additional 72 h (kifunensine was added to the plates at the time of PD-1 addition, until the end of PD-1 stimulation). Following these treatments, cells were plated in transwells, as described above.

In all invasion experiments, cells that migrated to the lower part of the membranes were fixed by methanol, stained with Hemacolor (#111661; Merck, Kenilworth, NJ, USA), photographed using light microscopy and counted at multiple high-power fields (HPF).

### 2.7. ELISA Assays

MDA cells undergoing different treatments were plated at similar cell numbers, and 48-96 h later, cell supernatants were collected. When relevant, one day after cell plating, the cells were exposed to kifunensine or its vehicle control for 72 h, followed by exposure to PD-1 or its control for additional 72 h, ending with collection of cell supernatants.

CXCL8 levels were determined in cleared supernatants (by centrifugation) and ELISA analyses were performed, using the following antibodies and recombinant proteins (all from Peprotech, Rocky Hill, NJ, USA): CXCL8 coating antibodies: #500-P28; CXCL8 detecting antibodies: #500-P28BT; recombinant human CXCL8: #200-08. HRP-conjugated streptavidin (#016-030-084, Jackson Immunoresearch laboratories) and substrate TMB/E solution (#ES001, Millipore, Burlington, MA, USA) were added, the reaction was stopped by addition of 0.18 M H2SO4 and absorbance was measured at 450 nm.

### 2.8. Generation of Glycosylation Mutants

MCF-7 cells that over-express human WT-PD-L1 or PD-L1 variants mutated in N-linked glycosylation sites were generated in parallel to control vector cells (“Vector” cells) that underwent a similar procedure with a sham vector. Briefly, cells have been infected to express mCherry-pQCXIN plasmid (with a neomycin selection marker) to allow for intravital in vivo analyses. Following mCherry infection, the cells underwent retroviral infections with WT-PD-L1-pQCXIP or with S283A-PD-L1-pQCXIH [10]. Control “Vector” cells were derived by infection of mCherry-expressing MCF-7 cells with an empty pQCXIP vector or empty pQCXIH vector [10]. In parallel, in the current study, MCF-7 cells expressing mCherry were infected to express PD-L1 variants mutated at N-linked glycosylation sites, generated as described below.

To generate a similar system in MDA cells, the expression of endogenous PD-L1 was first knocked-out (KO) using the Alt-R CRISPR-Cas9 system (Integrated DNA Technologies, Coralville, IA, USA) to generate a pool of three clones. These cells were infected by the control vector (“Vector” cells), by WT-PD-L1-pQCXIP or by S283A-PD-L1-pQCXIH [10]. In parallel, MDA cells expressing mCherry were infected to express the N-linked glycosylation-mutated PD-L1 variants, generated as described below.

To generate the N35A-PD-L1 mutant, we used the PD-L1 (N35A)-sense primer GTAGAGTATGGTAGCGCTATGACAATTGAATGC and the PD-L1 (N35A)-anti-sense primer GCATTCAATTGTCATAGCGCTACCATACTCTAC. To generate the N192A-PD-L1 glycosylation mutant, we used the PD-L1 (N192A)-sense primer GAGGAGAAGCTTTTCGCTGTGACCAGCACACTG and the PD-L1 (N192A)-anti-sense primer CAGTGTGCTGGTCACAGCGAAAAGCTTCTCCTC. The N200A-PD-L1 glycosylation mutant was generated using PD-L1 (N200A)-sense primer AGCACACTGAGAATCGCCACAACAACTAATGAGA and PD-L1 (N200A)-anti-sense primer TCTCATTAGTTGTTGTGGCGATTCTCAGTGTGCT. The N219A-PD-L1 glycosylation mutant was generated using PD-L1 (N219A)-sense primer TTAGATCCTGAGGAAGCCCATACAGCTGAATTG and PD-L1 (N219A)-anti-sense primer CAATTCAGCTGTATGGGCTTCCTCAGGATCTAA. We also constructed a mutant that included all four mutations above (termed 4A-PD-L1). The generated fragments were digested with Age1 and Pac1 and ligated into the corresponding sites of pQCXIP vector (https://www.addgene.org/vector-database/3870/; accessed on 15 January 2023). By sequencing, we validated that the PCR product of WT-PD-L1 was identical to the published sequence (NM_014143) and that PD-L1 variants carried the desired mutations. Overall, five mutated N-linked glycosylation mutants were generated: N35A-PD-L1, N192A-PD-L1, N200A-PD-L1, N219A-PD-L1 and 4A-PD-L1.

Following infection with WT-PD-L1, N35A-PD-L1, N192A-PD-L1, N200A-PD-L1, N219A-PD-L1, 4A-PD-L1 vectors and sham vector, PD-L1 expression was determined in MCF-7 cells and MDA cells by flow cytometry.

### 2.9. Tumor Growth and Metastasis

Three types of mCherry-expressing cells were administered orthotopically to the mammary fat pads of female athymic nude mice (#NUDE242; EnvigoRMS, Jerusalem, Israel) in two independent experimental repeats that were performed in a similar manner. The mice groups included mice injected with: WT-PD-L1-MDA cells (n = 10 mice in total), N35A-PD-L1-MDA cells (n = 10 mice in total) and N219A-PD-L1-MDA cells (n = 9 mice in total). The different cell types were mixed with matrigel (#354234; Sigma-Aldrich) at a 1:1 ratio prior to injection. Mice weight and tumor volumes were determined every 3–4 days by scale and caliper, respectively.

Since the mice groups had different lag periods in appearance of primary tumors, animals in each group of mice were sacrificed when tumor volumes in that group reached the size limits dictated by the regulations of the Ethics Committee for Animal Use at Tel Aviv University. Tumor volumes were determined during the process by using caliper, and were measured ex vivo when they were removed from mice. At the time of experiment termination, metastases were determined ex vivo in excised organs (lymph nodes that were adjacent to the primary tumors, lungs, liver and femur), based on mCherry signals, using the Maestro imaging device.

Comparisons between tumor volumes were performed by Anova. Statistical analyses of kinetics of tumor appearance were performed through Gehan–Breslow–Wilcoxon test. The proportions of mice bearing metastases were compared by Chi square test for trend.

Procedures involving experimental animals were approved by the Ethics Committee for Animal Use of Tel Aviv University (Approval no. 2306-138-5) and were performed in compliance with local animal welfare laws, guidelines and policies.

### 2.10. Analyses of Patient Datasets

The TCGA dataset [28] was used to perform survival analyses based on the abundance of MAN2B1 (coding for α-mannosidase I) and MAN2A1 (coding for α-mannosidase II). Kaplan–Meier analyses of overall survival and progression-free intervals were performed for each of the two genes based on their median expression levels. Disease subtypes were defined based on the dataset PAM50Call_RNAseq annotation file of the TCGA dataset, including 420 Luminal A patients and 140 basal-like patients (often overlapping with the term TNBC). Log rank tests, provided by the TCGA dataset, were used to determine *p* values.

### 2.11. Statistical Analyses

Statistical analyses of in vitro studies were performed by two-tailed unpaired Student’s *t*-tests. Statistical analyses of TCGA and in vivo studies were described in the relevant sections. Values of *p* ≤ 0.05 were considered statistically significant.

## 3. Results

### 3.1. The Cell-Autonomous and PD-1-Induced Pro-Metastatic Effects of PD-L1 in Breast Cancer Cells Are Mediated via STAT3 and STAT1 Activation

To address the possible involvement of the STAT3 and STAT1 pathways in regulating PD-L1 activities, we used similar research systems to those included in our published study [10]. The research systems included human luminal A breast cancer MCF-7 cells and human TNBC breast cancer MDA-MB-231 (MDA) cells. In MCF-7 cells—which do not express PD-L1 endogenously (Figure S1A)—we compared between cells infected to express WT-PD-L1 or S283A-PD-L1, and vector-infected cells (herein called “Vector” cells). In parallel, we used MDA cells in which the endogenous expression of PD-L1 was first knocked-out (KO) by CRISPR-Cas9; then, the cells were infected to express WT-PD-L1 or S283A-PD-L1 (Figure S1B); sham vectors were used as controls (“Vector” cells). The setup of both systems is described in our published study [10], and is also demonstrated for readers’ convenience again in Figure S1 (Figure S1 demonstrates different experiments than the published study; PD-L1 expression by Vector-MCF-7 cells and Vector-MDA cells is demonstrated in the next figures, below).

**Figure 1:**
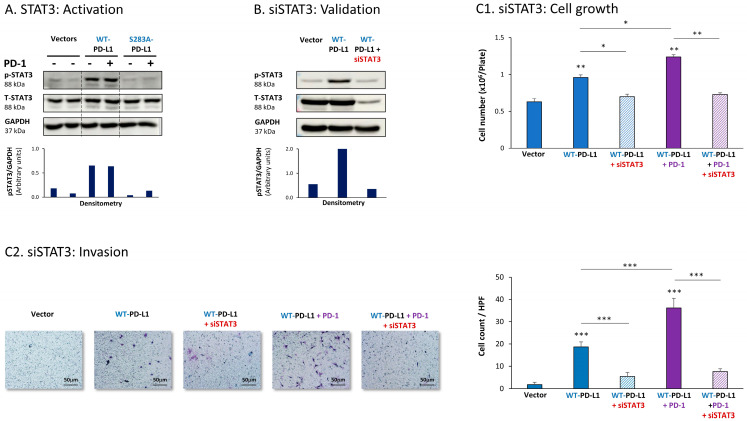
The cell-autonomous and PD-1-induced pro-metastatic activities of PD-L1 in MCF-7 cells are mediated by STAT3 activation. (**A**) STAT3 activation in human luminal A MCF-7 cells. WT-PD-L1-MCF-7 cells and S283A-PD-L1-MCF-7 cells (generated as described in Figure S1A) were exposed to PD-1 or its control for 72 h. Vector control MCF-7 cells (“Vector” cells, corresponding to WT-PD-L1 or S283A-PD-L1 cells) were treated with the control of PD-1 for the same time length. STAT3 activation (at position Y705) was determined at termination of PD-1 exposure (or its control) by WB analysis, using GAPDH as loading control. Densitometry results demonstrate arbitrary units of STAT3 activation values (pSTAT3/GAPDH) obtained in the different lanes of the corresponding WB figure. (**B**) Validation of siSTAT3 efficacy. WT-PD-L1-MCF-7 cells and Vector-MCF-7 cells were transiently transfected with siSTAT3 or siCTRL for 72 h. Then, down-regulation of STAT3 expression was determined by WB analyses, using GAPDH as loading control. Densitometry results demonstrate arbitrary units of STAT3 activation values (pSTAT3/GAPDH) obtained in the different lanes of the corresponding WB figure. (**C**) The effects of STAT3 down-regulation on PD-L1 functions. WT-PD-L1-MCF-7 cells and Vector-MCF-7 cells were transfected with siSTAT3 or its siCTRL for 72 h. Then, the cells were exposed to PD-1 or its control for additional 72 h, followed by analyses of cell functions. (**C1**) Cell growth, determined by cell counts. (**C2**) Tumor cell invasion, determined in transwells. HPF—High-power field. Size bar, 50 μm. *** *p* < 0.001, ** *p* < 0.01, * *p* < 0.05. Asterisks above bars denote statistical significance compared with Vector-MCF-7 cells. In panels A and C, the results are from a representative experiment of n = 3, showing similar results.

The expression of WT-PD-L1 by MCF-7 cells led to strong activation of STAT3, when WT-PD-L1-MCF-7 cells were compared to Vector-MCF-7 cells (Figure 1A). Moreover, STAT3 activation was almost completely abolished when PD-L1 was mutated to the non-functional S283A-PD-L1 variant, reducing STAT3 activation levels to those noted in Vector cells (Figure 1A). Exposing WT-PD-L1-MCF-7 cells to PD-1 has not induced an additional increase in STAT3 activation (Figure 1A), suggesting that the expression of PD-L1 by itself has led to high and saturated levels of STAT3 activation.

To explore the roles of STAT3 in mediating PD-L1 activities, we used siRNA to STAT3 (siSTAT3) (validated in Figure 1B), which was compared with siCTRL. The data of Figure 1C1 demonstrate that in MCF-7 cells, siSTAT3 has reduced the ability of WT-PD-L1 to up-regulate tumor cell growth. The ability of PD-1 stimulation to further elevate the growth of WT-PD-L1-expressing cells was also inhibited by siSTAT3 (Figure 1C1). In parallel, the increased invasion properties that were gained by MCF-7 cells due to the cell-autonomous and PD-1-induced activities of PD-L1 were also prominently decreased upon STAT3 down-regulation in WT-PD-L1-MCF-7 cells by siSTAT3 (Figure 1C2). CXCL8 was not determined in MCF-7 cells because of its very low expression levels in these cells.

**Figure 2:**
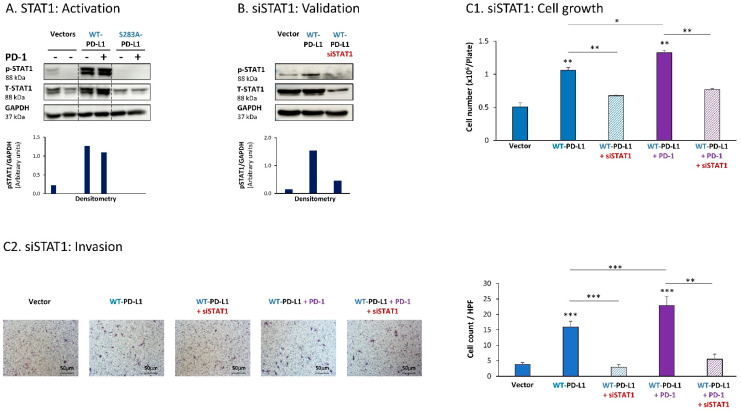
The cell-autonomous and PD-1-induced pro-metastatic activities of PD-L1 in MCF-7 cells are mediated by STAT1 activation. (**A**) STAT1 activation in human luminal A MCF-7 cells. WT-PD-L1-MCF-7 cells and S283A-PD-L1-MCF-7 cells (generated as described in Figure S1A) were exposed to PD-1 or its control for 72 h. Vector control MCF-7 cells (“Vector” cells; corresponding to WT-PD-L1 or S283A-PD-L1 cells) were treated with the control of PD-1 for the same time length. STAT1 activation was determined at termination of PD-1 exposure (or its control) by WB analysis, using GAPDH as loading control. Densitometry results demonstrate arbitrary units of STAT1 activation values (pSTAT1/GAPDH) obtained in the different lanes of the corresponding WB figure. (**B**) Validation of siSTAT1 efficacy. WT-PD-L1-MCF-7 cells and Vector-MCF-7 cells were transiently transfected with siSTAT1 or siCTRL for 72 h. Then, down-regulation of STAT1 expression was determined by WB analyses, using GAPDH as loading control. Densitometry results demonstrate arbitrary units of STAT1 activation values (pSTAT1/GAPDH) obtained in the different lanes of the corresponding WB figure. (**C**) The effects of STAT1 down-regulation on PD-L1 functions. WT-PD-L1-MCF-7 cells and Vector-MCF-7 cells were transfected with siSTAT1 or its siCTRL for 72 h. Then, the cells were exposed to PD-1 or its control for additional 72 h, followed by analyses of cell functions. (**C1**) Cell growth, determined by cell counts. (**C2**) Tumor cell invasion, determined in transwells. HPF—High-power field. Size bar, 50 μm. *** *p* < 0.001, ** *p* < 0.01, * *p* < 0.05. Asterisks above bars denote statistical significance compared with Vector-MCF-7 cells. In panels A and C, the results are from a representative experiment of n = 3, showing similar results.

Further analyses indicated that the cell-autonomous as well as the PD-1-induced activities of PD-L1 in MCF-7 cells also depended on STAT1 activation. Activation of STAT1 was clearly noted in WT-PD-L1-MCF-7 cells compared to Vector-MCF-7 cells, along with increased STAT1 expression levels (Figure 2A); STAT1 activation was not similarly elevated in cells expressing the inactive S283A-PD-L1 variant (Figure 2A). As with STAT3, PD-1 stimulation did not increase STAT1 activation any further, suggesting that not only STAT3, but also STAT1, has reached saturated levels of activation upon PD-L1 expression in the cells.

Following these observations, siSTAT1 (validated in Figure 2B) was found to prominently down-regulate the ability of PD-L1—in its cell-autonomous and PD-1-induced functions—to induce the pro-metastatic functions of cell growth and invasion of the cells (Figure 2C1,C2, respectively).

**Figure 3:**
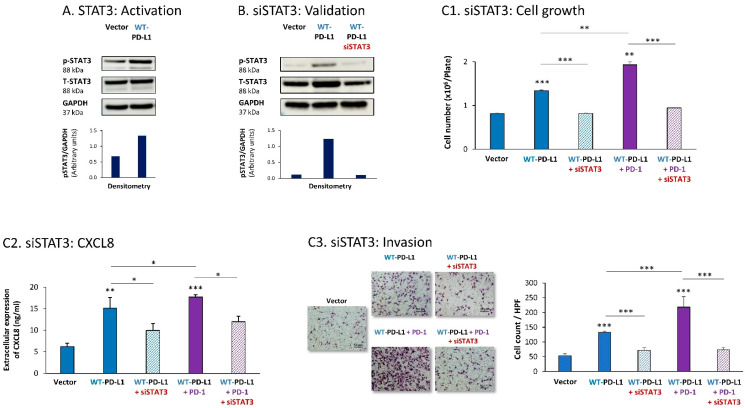
The cell-autonomous and PD-1-induced pro-metastatic activities of PD-L1 in MDA cells are mediated by STAT3 activation. (**A**) STAT3 activation in human TNBC MDA-MB-231 cells (MDA). STAT3 activation (at position Y705) was determined in WT-PD-L1-MDA cells compared with Vector-MDA cells (generated as described in Figure S1B) 48 h after plating, by WB analysis, using GAPDH as loading control. Densitometry results demonstrate arbitrary units of STAT3 activation values (pSTAT3/GAPDH) obtained in the different lanes of the corresponding WB figure. (**B**) Validation of siSTAT3 efficacy. WT-PD-L1-MDA cells and Vector-MDA cells were transiently transfected with siSTAT3 or siCTRL for 72 h. Then, down-regulation of STAT3 expression was determined by WB analyses, using GAPDH as loading control. Densitometry results demonstrate arbitrary units of STAT3 activation values (pSTAT3/GAPDH) obtained in the different lanes of the corresponding WB figure. (**C**) The effects of STAT3 down-regulation on PD-L1 functions in MDA cells. WT-PD-L1-MDA cells and Vector-MDA cells were transfected with siSTAT3 or its siCTRL for 72 h. Then, the cells were exposed to PD-1 or its control for additional 72 h, followed by analyses of cell functions. (**C1**) Cell growth, determined by cell counts. (**C2**) CXCL8 production, determined by ELISA. (**C3**) Tumor cell invasion, determined in transwells. HPF—High-power field. Size bar, 50 μm. *** *p* < 0.001, ** *p* < 0.01, * *p* < 0.05. Asterisks above bars denote statistical significance compared to Vector- MDA cells. In panels A and C, the results are from a representative experiment of n = 3, showing similar results.

Parallel experiments that were performed with MDA cells provided additional evidence for the key roles played by STAT3 and STAT1 in mediating PD-L1 functions in breast cancer cells. First, we found that STAT3 was activated in WT-PD-L1-MDA cells compared with Vector-MDA cells (Figure 3A) (due to technical inconsistencies noted in multiple experiments that were performed at a wide range of conditions, we could not conclude whether STAT3 activation was further elevated by PD-1 stimulation or was reduced in cells expressing the S283A-PD-L1 variant). A clear-cut role was revealed for STAT3 in regulating the cell-autonomous and pro-metastatic functions of PD-L1 in MDA cells, as indicated by the ability of siSTAT3 (validated in Figure 3B) to reduce the ability of PD-L1 to induce MDA cell growth and to reduce CXCL8 release by MDA cells as well as their invasion (Figure 3C1–C3). Inhibitory effects of siSTAT3 on these tumor cell functions were also noted upon PD-1 stimulation (Figure 3C1–C3).

**Figure 4:**
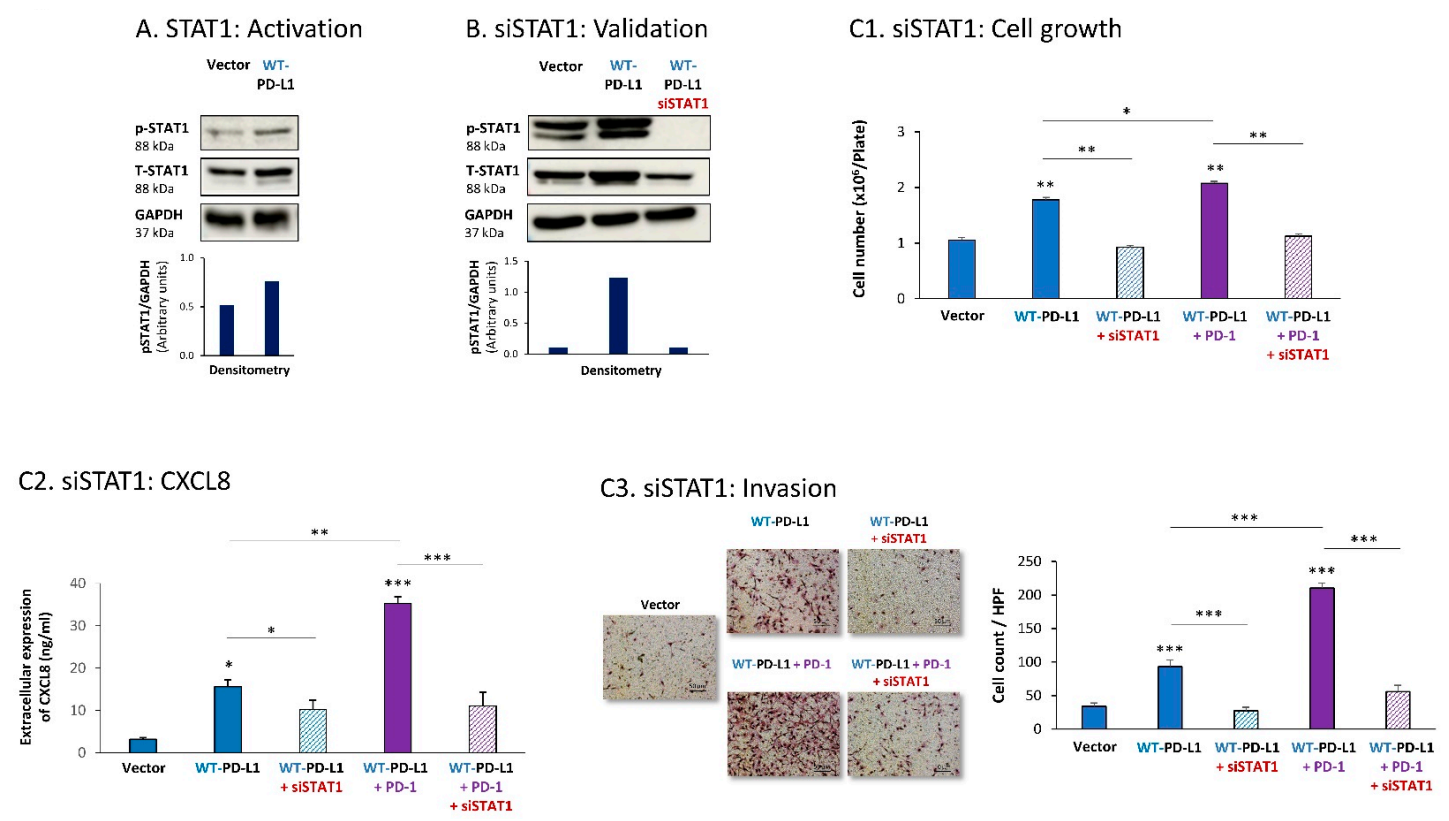
The cell-autonomous and PD-1-induced pro-metastatic activities of PD-L1 in MDA cells are mediated by STAT1 activation. (**A**) STAT1 activation in human TNBC MDA-MB-231 cells (MDA). STAT1 activation was determined in WT-PD-L1-MDA cells compared with Vector-MDA cells (generated as described in Figure S1B) 48 h after plating, by WB analysis, using GAPDH as loading control. Densitometry results demonstrate arbitrary units of STAT1 activation values (pSTAT1/GAPDH) obtained in the different lanes of the corresponding WB figure. (**B**) Validation of siSTAT1 efficacy. WT-PD-L1-MDA cells and Vector-MDA cells were transiently transfected with siSTAT1 or siCTRL for 72 h. Then, down-regulation of STAT1 expression was determined by WB analyses, using GAPDH as loading control. Densitometry results demonstrate arbitrary units of STAT1 activation values (pSTAT1/GAPDH) obtained in the different lanes of the corresponding WB figure. (**C**) The effects of STAT1 down-regulation on PD-L1 functions in MDA cells. WT-PD-L1-MDA cells and Vector-MDA cells were transfected with siSTAT1 or its siCTRL for 72 h. Then, the cells were exposed to PD-1 or its control for additional 72 h, followed by analyses of cell functions. (**C1**) Cell growth, determined by cell counts. (**C2**) CXCL8 production, determined by ELISA. (**C3**) Tumor cell invasion, determined in transwells. HPF—High-power field. Size bar, 50 μm. *** *p* < 0.001, ** *p* < 0.01, * *p* < 0.05. Asterisks above bars denote statistical significance compared to Vector- MDA cells. In panels A and C, the results are from a representative experiment of n = 3, showing similar results.

Then, STAT1 activation was also found to be essential for PD-L1-induced pro-metastatic functions in MDA cells. In this case, we found that expression of WT-PD-L1 by the cells had led to STAT1 activation (Figure 4A) (as noted for STAT3, here, we could also not ascertain the impact of PD-1 stimulation or the S283A-PD-L1 variant on STAT1 activation, due to experimental inconsistencies). STAT1 down-regulation by siSTAT1 (validated in Figure 4B) demonstrated that STAT1-mediated activities were required for PD-L1 to exert cell-autonomous and PD-1-induced functions in MDA cells. This was manifested by reduced tumor cell growth, CXCL8 release and invasion in siSTAT1-expressing cells compared with siCTRL-expressing cells in WT-PD-L1 cells, not treated or treated with PD-1 (Figure 4C1–C3).

Together, the findings in this part of the study indicate that both STAT3 and STAT1 play key roles in mediating the cell-autonomous and PD-1-induced pro-metastatic functions of PD-L1 in breast cancer cells.

### 3.2. Inhibition of N-Linked Glycosylation Leads to Reduced Pro-Metastatic Potential of PD-L1 in Breast Cancer Cells In Vitro

To determine the roles of N-linked glycosylation in regulating PD-L1 pro-metastatic activities in breast cancer cells, we used kifunensine (K) and swainsonine (S), which are inhibitors of key enzymes participating in N-linked glycosylation: α-mannosidase I and α-mannosidase II, respectively [29,30]. As described below, we analyzed the effects of the inhibitors on the phenotype of PD-L1 glycosylation, on STAT3 and STAT1 activation and on PD-L1-driven pro-metastatic functions in MCF-7 cells and MDA cells (STAT3 and STAT1 activation was determined only in MCF-7 cells).

**Figure 5:**
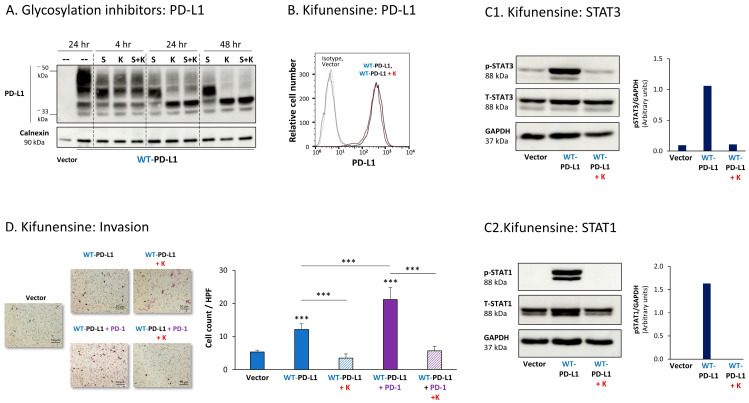
Inhibition of N-linked glycosylation leads to reduced PD-L1 glycosylation and to lower PD-L1-induced pro-metastatic effects in MCF-7 cells, in vitro. (**A**) The effects of N-linked glycosylation inhibitors on PD-L1 glycosylation in WT-PD-L1-MCF-7 cells. The cells were treated with kifunensine (termed “K”; 50 μM) and/or swainsonine (termed “S”; 50 μM) for different time points (compared with their vehicle control at 24 h), as demonstrated in the figure. PD-L1 expression was determined by WB, using calnexin as loading control. (**B**) PD-L1 cell surface expression in WT-PD-L1-MCF-7 cells, treated by kifunensine or its vehicle, and in vehicle-treated Vector-MCF-7 cells. PD-L1 expression was determined by flow cytometry. Non-relevant isotype-matched antibodies were used as an isotype control. (**C1**,**C2**) The effects of kifunensine on STAT3 (**C1**) and STAT1 (**C2**) activation. The activation of the STAT3 (at the Y705 position) and of STAT1 was determined in WT-PD-L1-MCF-7 cells treated with kifunensine or its vehicle (48 h), and in Vector-MCF-7 cells that were treated with the vehicle. STAT3 and STAT1 activation was determined by WB analysis, using GAPDH as loading control. Densitometry results demonstrate arbitrary units of STAT3 activation values (pSTAT3/GAPDH) and of STAT1 activation values (STAT1/GAPDH) obtained in the different lanes of the corresponding WB figures. (**D**) The effects of kifunensine on invasion of WT-PD-L1-MCF-7 cells. Invasion was determined in WT-PD-L1-MCF-7 cells treated by kifunensine or its vehicle, and in vehicle-treated Vector-MCF-7 cells, in transwells. HPF—High-power field. Size bar, 50 μm. *** *p* < 0.001. Asterisks above bars denote statistical significance compared with Vector-MCF-7 cells. In all panels, the results are from a representative experiment of n = 3, showing similar results.

Figure 5A demonstrates that both kifunensine and swainsonine have reduced PD-L1 N-linked glycosylation in MCF-7 cells in a time-dependent manner, reaching maximal effect at the 24 h to 48 h time points. Prior to treatment by the inhibitors, WT-PD-L1 demonstrated a large range of molecular weights (MWs) due to its varied levels of glycosylation. Following cell treatment by kifunensine and somewhat less by swainsonine, PD-L1 has gained a much narrower dispersion in WB; its MW was much reduced, and it almost reached the ~33 kDa expected for the non-glycosylated protein (Figure 5A).

Due to its more prominent effects on PD-L1 N-linked glycosylation, the effects of kifunensine on the pro-tumorigenic functions of PD-L1 were determined. After verifying that the expression levels of PD-L1 at the cell membrane remained intact following kifunensine treatment (Figure 5B), we found that the inhibitor has almost completely reduced the PD-L1-induced elevation in STAT3 and STAT1 activation in WT-PD-L1-expressing cells (Figure 5C). Moreover, kifunensine has markedly reduced the cell-autonomous and PD-1-induced abilities of PD-L1 to promote tumor cell invasion (Figure 5D; cell proliferation was not affected by kifunensine).

**Figure 6:**
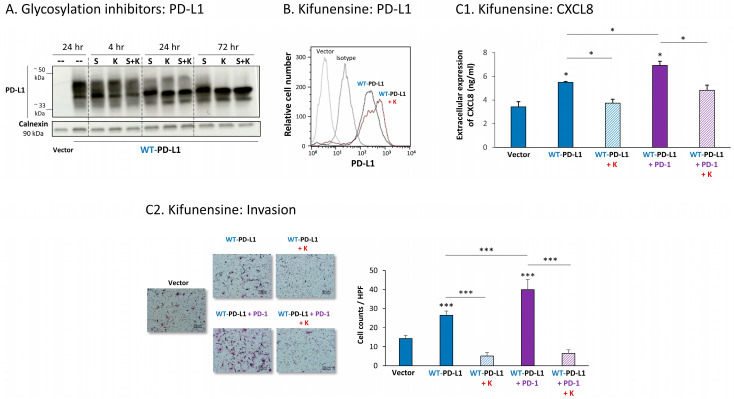
Inhibition of N-linked glycosylation leads to reduced PD-L1 glycosylation and to lower PD-L1-induced pro-metastatic effects in MDA cells, in vitro. (**A**) The effects of N-linked glycosylation inhibitors on PD-L1 glycosylation in WT-PD-L1-MDA cells. The cells were treated with kifunensine (termed “K”; 50 μM) and/or swainsonine (termed “S”; 50 μM) for different time points (compared with vehicle control at 24 h), as demonstrated in the figure. PD-L1 expression was determined by WB, using calnexin as loading control. (**B**) PD-L1 cell surface expression in WT-PD-L1-MDA, treated with kifunensine or its vehicle, and in vehicle-treated Vector-MDA cells. PD-L1 expression was determined by flow cytometry. Non-relevant isotype-matched antibodies were used as an isotype control. (**C**) The effects of kifunensine on functions of WT-PD-L1-MDA cells. Cell functions were determined in WT-PD-L1-MDA cells treated with kifunensine or its vehicle (72 h). (**C1**) CXCL8 production, determined by ELISA. (**C2**) Tumor cell invasion, in transwells. HPF—High-power field. Size bar, 50 μm. *** *p* < 0.001, * *p* < 0.05. Asterisks above bars denote statistical significance compared with Vector-MDA cells. In panels A and C, the results are from a representative experiment of n = 3, and in panel B of n = 2, showing similar results.

Parallel analysis of MDA cells demonstrated similar results, indicating that the inhibition of N-linked glycosylation of WT-PD-L1 was connected to reduced pro-metastatic effects of PD-L1. First, we found that the N-linked glycosylation of PD-L1 was substantially reduced by kifunensine and swainsonine, clearly seen after 72 h of their use (Figure 6A). The cell surface expression of WT-PD-L1 remained intact when MDA cells were treated with kifunensine (Figure 6B), yet the ability of PD-L1 to promote CXCL8 release and invasion of the tumor cells in cell-autonomous and PD-1-induced manners was prominently reduced upon kifunensine treatment (Figure 6C; cell proliferation was not affected by kifunensine).

Together, these findings indicate that down-regulation of PD-L1 N-linked glycosylation was connected to inhibition of STAT3 and STAT1 activation (noted in MCF-7 cells), and to reduced cell-autonomous and PD-1-induced tumor cell invasion and release of pro-tumorigenic factors in breast cancer cells. These findings suggest that N-linked glycosylation of PD-L1 is required for its abilities to induce pro-metastatic functions of breast cancer cells, which were mediated by STAT3 and STAT1 activation.

### 3.3. N-Linked Glycosylation of PD-L1 Is Required for Its Cell-Autonomous Pro-Metastatic In Vitro Functions, and for STAT3 and STAT1 Activation in Breast Cancer Cells

To further explore the roles of PD-L1 N-linked glycosylation in regulating its functional effects in breast cancer cells, each of its four N-linked glycosylation sites (N35, N192, N200 and N219; [25,26,27]) was separately mutated, and a mutant of all four sites combined was also generated. As with WT-PD-L1-expressing cells, the cell systems that were generated were based on MCF-7 cells and MDA cells. In MCF-7 cells, each of the mutated PD-L1 constructs was individually expressed and was compared to WT-PD-L1-MCF-7 cells and to Vector-MCF-7 cells. In MDA cells, the mutated PD-L1 constructs were infected to cells in which the endogenous PD-L1 was first knocked-out (as previously described and demonstrated in Figure S1B), and were tested in parallel with WT-PD-L1-MDA and Vector-MDA cells (generated in a similar manner, as described before). Overall, the MCF-7 and MDA cell systems included WT-PD-L1 cells, N35A-PD-L1 cells, N192A-PD-L1 cells, N200A-PD-L1 cells, N219A-PD-L1 cells, 4A-PD-L1 cells and Vector-expressing control cells.

**Figure 7:**
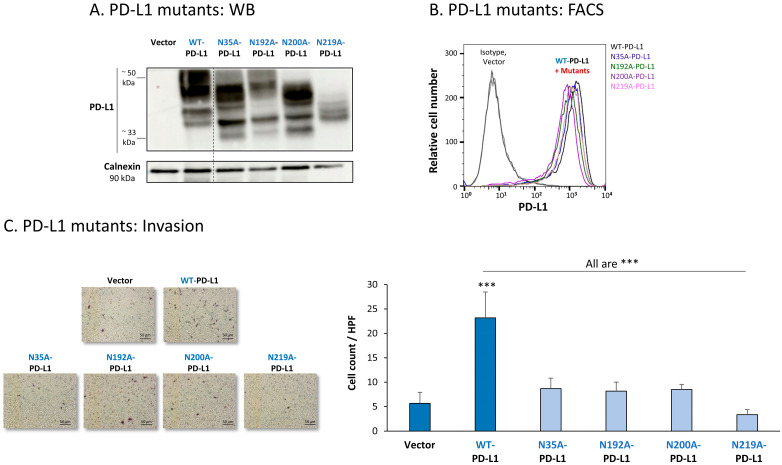
The in vitro pro-metastatic effects of PD-L1 in MCF-7 cells require N-linked glycosylation of PD-L1 at all four sites. The phenotypes and functions of MCF-7 cells expressing WT-PD-L1 or PD-L1 variants, mutated singly at each of the four N-linked glycosylation sites (N35A-PD-L1, N192A-PD-L1, N200A-PD-L1 and N219A-PD-L1) were determined in parallel with Vector-MCF-7 cells. (**A**) PD-L1 expression, determined by WB using calnexin as loading control. (**B**) PD-L1 cell surface expression, determined by flow cytometry (FACS). Non-relevant isotype-matched antibodies were used as an isotype control. (**C**) Tumor cell invasion, in transwells. HPF—High-power field. Size bar, 50 μm. *** *p* < 0.001. Asterisks above bar denote statistical significance compared with Vector-MCF-7 cells. In all panels, the results are from a representative experiment of n = 3, showing similar results.

The findings of Figure 7A indicate that the glycosylation pattern of PD-L1 was disrupted in each of the four MCF-7 cell types expressing a single mutation in N-linked glycosylation sites of the protein. The dispersion of PD-L1 due to glycosylation was reduced the most in cells expressing the N219A-PD-L1 mutant (Figure 7A). In parallel, in cells expressing the N35A-PD-L1 and the N200A-PD-L1 mutants, PD-L1 demonstrated a substantial shift towards the low MW range, and a similar dispersion pattern. A modified glycosylation pattern was also revealed for the N192A-PD-L1 variant, but it was somewhat less prominent than for N35A-PD-L1 and N200A-PD-L1. Of note, the MW of the 4A-PD-L1 mutant—in which all four N-glycosylation sites were mutated—was ~33 kDa, as expected (Figure S2A1).

All four single mutants—N35A-PD-L1, N192A-PD-L1, N200A-PD-L1 and N219A-PD-L1—were expressed by MCF-7 cells at normal levels, similar to WT-PD-L1 (Figure 7B) (the 4A-PD-L1 mutant was detected less by flow cytometry compared with WT-PD-L1 (Figure S2B1), therefore, we did not study the 4A-PD-L1 variant any further). Yet, each and every one of the cells expressing these separate mutants demonstrated impaired invasion capacities when they were compared with WT-PD-L1 MCF-7 cells (Figure 7C). The strongest reduction in invasion was noted for MCF-7 cells expressing the N219A-PD-L1 mutant.

**Figure 8:**
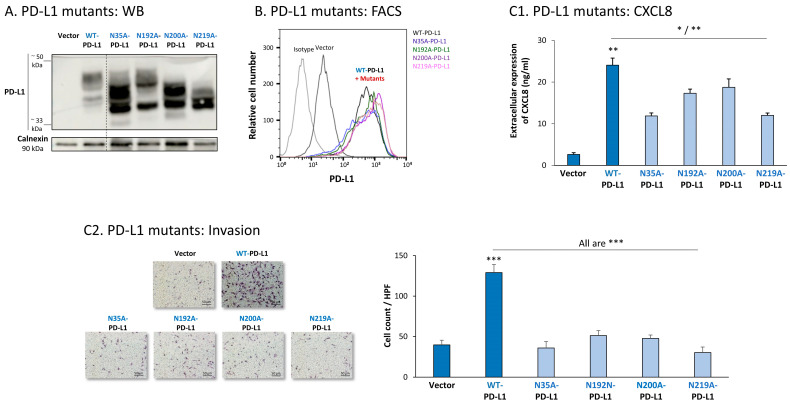
The in vitro pro-metastatic effects of PD-L1 in MDA cells require N-linked glycosylation of PD-L1 at all four sites. The phenotypes and functions of MDA cells expressing WT-PD-L1 or PD-L1 variants, mutated singly at each of the four N-linked glycosylation sites (N35A-PD-L1, N192A-PD-L1, N200A-PD-L1 and N219A-PD-L1) were determined in parallel with Vector-MDA cells. (**A**) PD-L1 expression, determined by WB using calnexin as loading control. (**B**) PD-L1 cell surface expression, determined by flow cytometry (FACS). Non-relevant isotype-matched antibodies were used as an isotype control. (**C**) Tumor cell functions. (**C1**) CXCL8 production, determined by ELISA. (**C2**) Tumor cell invasion, in transwells. HPF—High-power field. Size bar, 50 μm. *** *p* < 0.001, ** *p* < 0.01, * *p* < 0.05. Asterisks above bar denote statistical significance compared with Vector-MDA cells. In all panels, the results are from a representative experiment of n = 3, showing similar results.

The study of MDA cells has provided further support to the roles of each of the four N-linked glycosylation sites of PD-L1 in regulating its pro-metastatic impacts on the cancer cells. The dispersion patterns of the N-linked glycosylation mutants in MDA cells by WB (Figure 8A) were in general similar to those noted in MCF-7 cells (Figure 7A), demonstrating the strongest reduction in PD-L1 glycosylation in the N219A-PD-L1-expressing cells (Figure 8A). Here again, the 4A-PD-L1 mutant reached the expected ~33 kDa of the non-glycosylated protein (Figure S2A2).

Each of the four N-linked glycosylation single mutants was normally expressed by MDA cells, at similar levels to WT-PD-L1 (Figure 8B), while the 4A-PD-L1 was not detected at levels that were above those of endogenous PD-L1 expression (Figure S2B2). Despite their intact expression at the cell surface, all single N-linked glycosylation mutants had a significantly reduced ability to induce CXCL8 expression in the tumor cells, as well as cancer cell invasion (Figure 8(C1,C2), respectively). Mutations at the N35 and N219 sites had the most substantial impact on CXCL8 production and cell motility in MDA cells.

**Figure 9:**
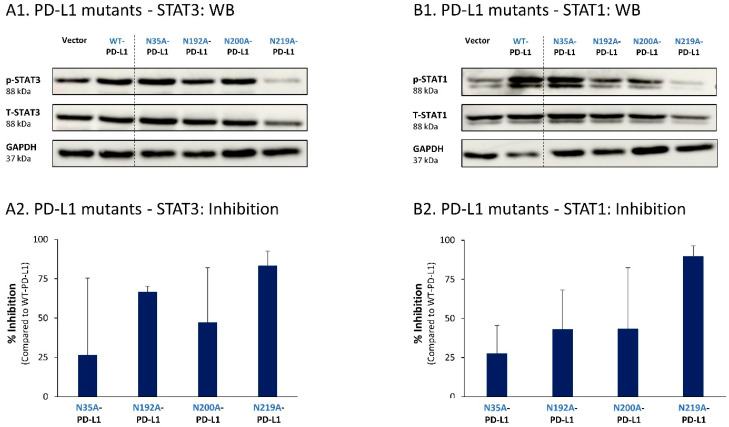
The N-linked glycosylation of PD-L1, mainly at the N219 site, is required for PD-L1-induced STAT3 and STAT1 activation in MCF-7 cells. STAT3 (**A**) and STAT1 (**B**) activation were determined in MCF-7 cells expressing WT-PD-L1 or PD-L1 variants mutated singly at each of the PD-L1 N-linked glycosylation sites (N35A-PD-L1, N192A-PD-L1, N200A-PD-L1 and N219A-PD-L1), in parallel with Vector-MCF-7 cells. STAT3 and STAT1 activation was determined by WB analyses, using GAPDH as loading control. (**A1**,**B1**) WB figures, demonstrating STAT3 (**A1**) and STAT1 (**B1**) activation, determined 48 h after MCF-7 cell culturing. In both panels, the results are from a representative experiment of n = 3, generally showing similar results. (**A2**,**B2**) % inhibition of STAT3 activation (**A2**) and of STAT1 activation (**B2**), determined based on arbitrary units of their activation in each PD-L1 variant compared with WT-PD-L1 (more details are provided in Section 2). The data presented in each bar are the average ± SD of % inhibition, obtained in n > 3 experiments.

To follow up on our previous findings demonstrating the key roles of STAT3 and STAT1 in mediating the cell-autonomous pro-metastatic effects of PD-L1 in breast cancer cells, we next determined the impact of each of the four N-linked glycosylation sites on STAT3 and STAT1 activation. The findings of Figure 9 indicate that the activation of STAT3 and STAT1 in MCF-7 cells was impaired by >75% when PD-L1 was mutated at the N219 N-linked glycosylation site. Thus, N-linked glycosylation of the N219 site had the most substantial role in controlling PD-L1-induced activation of both STAT3 and STAT1 (Figure 9A,B, respectively); they were joined by the involvement of the N192 site in regulating PD-L1-induced STAT3 activation (Figure 9A). The N200 glycosylation site of PD-L1 demonstrated a less stable effect in determining PD-L1-induced STAT3 and STAT1 activation in MCF-7 cells, and minor roles were revealed for the N35 site (Figure 9).

**Figure 10:**
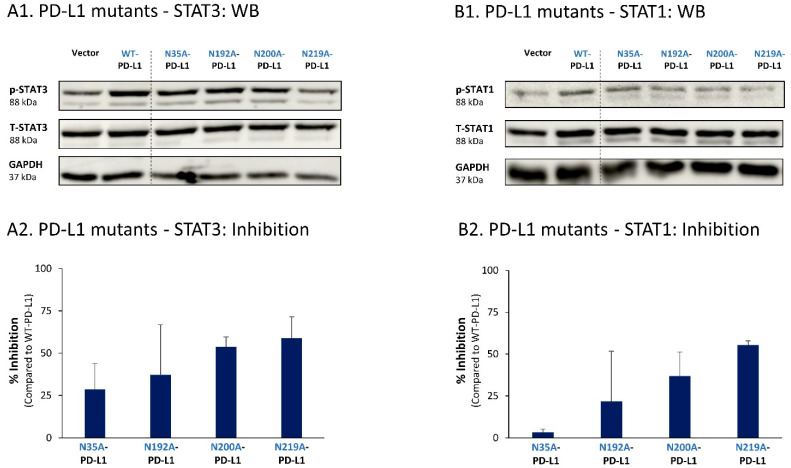
The N-linked glycosylation of PD-L1, mainly at the N219 site, is required for PD-L1-induced STAT3 and STAT1 activation in MDA cells. STAT3 (**A**) and STAT1 (**B**) activation were determined in MDA cells expressing WT-PD-L1 or PD-L1 variants mutated singly at each of the PD-L1 N-linked glycosylation sites (N35A-PD-L1, N192A-PD-L1, N200A-PD-L1 and N219A-PD-L1), in parallel with Vector-MDA cells. STAT3 and STAT1 activation was determined by WB analyses, using GAPDH as loading control. (**A1**,**B1**) WB figures, demonstrating STAT3 (**A1**) and STAT1 (**B1**) activation, determined 48 h after MDA cell culturing. In both panels, the results are from a representative experiment of n = 3, generally showing similar results. (**A2**,**B2**) % inhibition of STAT3 activation (**A2**) and of STAT1 activation (**B2**), determined based on arbitrary units of their activation in each PD-L1 variant compared with WT-PD-L1 (more details are provided in Section 2). The data presented in each bar are the average ± SD of % inhibition, obtained in n > 3 experiments.

Similar studies that were performed in MDA cells also pointed to N219 as the major site regulating PD-L1-induced STAT3 and STAT1 activation (Figure 10A,B, respectively). In MDA cells, the N200 glycosylation site of PD-L1 also plays a role in controlling STAT3 activation (Figure 10A), with less stable effects noted for the N192 site on PD-L1-induced STAT3 and STAT1 activation (Figure 10A,B). As with MCF-7 cells, in MDA cells, the N35 glycosylation site also had a minor role in this respect.

Thus, the findings of this part of the study indicate that the cell-autonomous pro-metastatic activities of PD-L1 in breast cancer cells require the glycosylation of each and every site of the four N-linked glycosylation sites of PD-L1. In parallel, it is mainly the N219 site whose glycosylation leads to STAT3 and STAT1 activation, with some roles also attributed to the N192 and N200 sites (depending on the cell type), consequently giving rise to pro-metastatic phenotypes in the cancer cells upon PD-L1 expression (as found in Figure 1, Figure 2, Figure 3 and Figure 4).

### 3.4. Impairment of Single N-Linked Glycosylation Sites of PD-L1 Reduces the Cell-Autonomous Pro-Metastatic Potential of Breast Tumor Cells In Vivo

To further elucidate the roles of N-linked glycosylation in regulating the cell-autonomous malignancy-promoting activities of PD-L1, we have used the TNBC in vivo model system based on MDA cells. Here, we determined tumor growth and metastasis of WT-PD-L1 cells, compared with cells in which PD-L1 was mutated at the N35 and N219 sites; these two sites were selected for the in vivo studies in view of the fact that they had the strongest involvement in regulating the cell-autonomous, pro-metastatic functions of PD-L1 in MDA cells in vitro (Figure 8).

**Figure 11:**
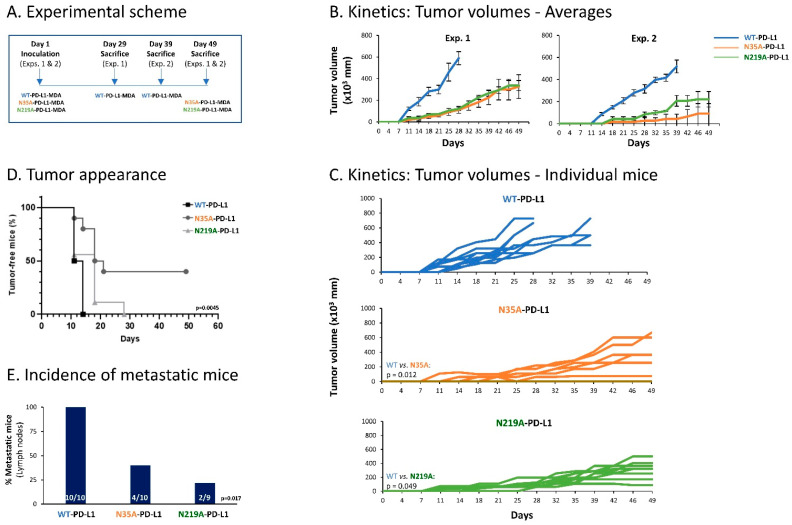
Impairment of single N-linked glycosylation sites of PD-L1 reduces the cell-autonomous, pro-metastatic activities of PD-L1 in vivo. mCherry-expressing MDA cells were administered to the mammary fat pad of female nude mice (deficient in T cell activities), in two independent experiments that were performed in a similar manner. The cells included WT-PD-L1-MDA cells (n = 10 mice in total: n = 5 in the first experiment and n = 5 in the second experiment), N35A-PD-L1-MDA (n = 10 mice in total: n = 6 in the first experiment and n = 4 in the second experiment) and N219A-PD-L1-MDA cells (n = 9 mice in total: n = 6 in the first experiment and n = 3 in the second experiment). (**A**) Experimental scheme. Mice of each group were sacrificed when reaching the humane point dictated by animal welfare laws. Tumor volumes were determined along the process using caliper and were measured ex vivo at time of experiment termination. Metastases were determined in different organs ex vivo, at the time that mice were sacrificed, based on mCherry signals. (**B**) Averages of tumor volumes in each of the mice groups, in the two independent experiments. (**C**) Schemes demonstrating tumor volumes in each of the mice included in both experiments together. p values are demonstrated in the figure. (**D**) Tumor cell appearance in mice from the three different groups (both experiments together). *p* = 0.0045 for comparison between all three groups (as demonstrated in the figure); *p* = 0.0012 for comparison between WT-PD-L1-MDA cells and N35A-PD-L1-MDA cells; *p* = 0.1881 for comparison between WT-PD-L1-MDA cells and N219A-PD-L1-MDA cells; *p* = 0.0486 for comparison between N35A-PD-L1-MDA cells and N219A-PD-L1-MDA cells. (**E**) Incidence of mice with lymph node metastases (in both experiments together); metastases in other organs (lungs, liver and femur) were much less frequent, and thus are not demonstrated. *p* values for comparisons between all three groups are demonstrated in the figure.

Thus, here we used WT-PD-L1-MDA, N35A-PD-L1-MDA and N219A-PD-L1-MDA cells, which were also infected to express mCherry, in order to allow for detection of metastases in remote organs by the Maestro imaging device. In two separate experiments that demonstrated similar results, the cells were administered to the mammary fat pad of female nude mice that are deficient in T cell activities. This approach has enabled us to analyze the roles of PD-L1 N-linked glycosylation sites in regulating the pro-malignancy effects of PD-L1 in vivo, independently of T cell activities, but rather through the cell-autonomous functions of PD-L1. In each mice group, tumors were excised when they had reached the humane time point indicated by animal welfare laws, and metastases were identified ex vivo at this time point in different organs by mCherry signals (an experimental scheme is provided in Figure 11A).

The findings of Figure 11 clearly indicate that impairment of PD-L1 N-linked glycosylation significantly reduced the tumorigenic and metastatic potential of the tumor cells. Kinetics and tumor volume analyses demonstrated a more rapid growth of WT-PD-L1-MDA cells than of N35A-PD-L1-MDA cells and N219A-PD-L1-MDA cells; also, mice injected with WT-PD-L1-expressing cells developed larger tumors than mice injected with N35A-PD-L1-MDA cells and N219A-PD-L1-MDA cells (Figure 11B–D). Metastases were detected mainly in lymph nodes adjacent to the mammary fat pad in which tumor cells were administered to the mice. The findings of Figure 11E indicate that the incidence of mice with lymph node metastases was significantly higher in mice injected with WT-PD-L1-MDA cells than in mice injected with N35A-PD-L1-MDA cells and N219A-PD-L1-MDA cells.

Overall, the findings of the in vivo studies indicate that in order to reach full potency in terms of tumor growth and metastasis, PD-L1 has to be N-linked glycosylated. In fact, the data also demonstrate that disruption of even one of the four N-linked glycosylation sites of PD-L1 can impair the tumorigenic cell-autonomous activities of PD-L1 in a mouse model.

### 3.5. High Levels of Enzymes Involved in N-Linked Glycosylation Are Associated with Reduced Survival in Breast Cancer Patients

The data presented in Figure 5 and Figure 6 indicated that kifunensine and swainsonine—inhibiting the mannose trimming steps taking place during the N-linked glycosylation process—lead to reduced N-linked glycosylation of PD-L1 in breast cancer cells. We have then demonstrated that the metastasis-promoting functions of PD-L1 depended on the protein being N-linked glycosylated. This was revealed not only by studies of tumor cell invasion and CXCL8 release, but also by in vivo analyses demonstrating the roles of PD-L1 N-linked glycosylation in determining the tumorigenic and metastatic activities of breast tumor cells.

**Figure 12:**
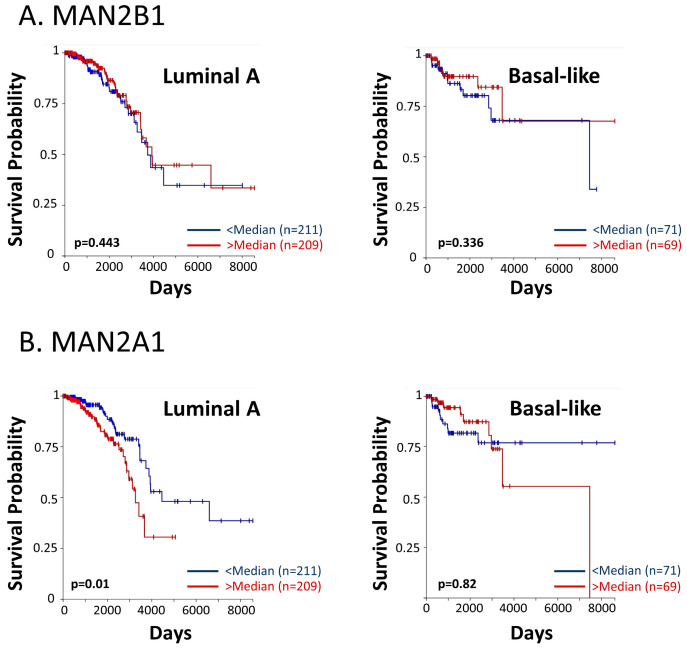
High levels of α-mannosidase II are associated with reduced survival in breast cancer patients. The TCGA dataset was used to determine the connection between patient overall survival and the expression levels of (**A**) α-mannosidase I (MAN2B1) and (**B**) α-mannosidase II (MAN2A1). In each patient group, “Luminal A” and “Basal-like”, the patient cohort was divided based on median values of gene expression (above median = “>median”; below median = “<median”). Patient numbers in each group and *p* values are indicated in the graphs.

These findings have led us to analyze the associations between patient survival and key enzymes that take part in the N-linked glycosylation process. Using the TCGA dataset, we analyzed the two mannose-trimming enzymes α-mannosidases I and II (inhibited by kifunensine and swainsonine, respectively), and their associations with overall survival of breast cancer patients of the luminal A and basal-like subtypes (in the PAM50 categorization, the basal-like subtype corresponds mainly to the TNBC subtype). The findings of Figure 12 provide evidence for significant associations between the expression levels of α-mannosidase II (MAN2A1) and patient overall survival in the luminal A subtype. A similar trend was revealed in the basal-like subtype (Figure 12), although not reaching statistical significance due to the low numbers of TNBC patients that were included in the dataset. For both breast cancer subtypes, the results of progression-free intervals are demonstrated in Figure S3.

To conclude, these findings connect a lower patient survival with increased levels of processes taking place in the course of N-linked glycosylation, and are in line with our other findings in this study, demonstrating key roles for PD-L1 N-linked glycosylation in increasing metastasis-promoting events, in vitro and in vivo.

## 4. Discussion

A major breakthrough in cancer therapy has been achieved by directing ICBs to the PD-L1/PD-1 axis, well recognized for its pivotal roles in shutting down potential T cell activities against cancer cells [1,2,3]. In parallel, it was recently reported that PD-L1 expression by cancer cells can promote pro-metastatic phenotypes and functions of the tumor cells [14,31,32,33,34].

Within this line of research, our recently published study has demonstrated that PD-L1 leads to pro-metastatic functions in breast cancer cells, which are further potentiated by PD-1 stimulation [10]. These activities, as well as the in vivo tumor- and metastasis-promoting effects of PD-L1 in a T cell-independent system, demonstrated that PD-L1 acts in a cell-autonomous manner to increase aggressiveness in breast cancer. Moreover, we found that all these activities were fully dependent on the integrity of the S283 intracellular residue of PD-L1 [10].

The roles of PD-L1 as an inducer of tumor cell properties that elevate tumor progression suggest that therapeutic strategies directed towards its immune-suppressive properties, alongside with measures inhibiting its cell-autonomous functions, should be considered in breast cancer therapy. Moreover, they emphasize the need to identify the mechanisms mediating and regulating PD-L1 functions in breast tumor cells.

Accordingly, in this study we have provided several novel findings on PD-L1 functions and regulation in breast cancer cells: (1) We have identified STAT3 and STAT1 as major molecular pathways mediating PD-L1 functions, cell-autonomous as well as PD-1-induced, in breast cancer cells; (2) we have shown that PD-L1 has to be N-linked glycosylated at all four sites in order to exert its pro-metastatic effects in breast cancer cells in vitro, at the levels of tumor cell invasion and CXCL8 release; (3) of the four different N-linked glycosylation sites, it was mainly the N219 site that induced STAT3 and STAT1 activation, with accompanying roles for the N192 and N200 N-linked glycosylation sites (depending on the cell type), leading to the cell-autonomous pro-metastatic functions of PD-L1 in vitro; (4) the malignancy potential of breast tumor cells in vivo was highly dependent on PD-L1 N-linked glycosylation; here, we are the first to show that the **cell-autonomous** tumor-promoting and metastatic potential of breast tumor cells was significantly reduced upon impairment of even one of the four N-linked glycosylation sites of PD-L1, e.g., N35 or N219.

To date, several studies have addressed the signaling pathways induced by PD-L1 in cancer cells, connecting mTOR/AKT activation with increased PD-L1 activities in cancer cells, including in breast tumor cells [11,12,13,14]. In this respect, it is interesting to note that interactions between the mTOR and the STAT pathways are known to take place in immune cells [15], suggesting that our observations on the roles of STAT3 and STAT1 in mediating PD-L1 activities in breast cancer may be connected to the activation of the mTOR axis. In this context, it is interesting to note that PD-1 stimulation of MCF-7 cells has led to increased pro-metastatic effects in the cells, without further elevating STAT3 or STAT1 activation. These findings support the possibility that STAT3 and STAT1 activation cooperates with additional signaling pathways that are induced by PD-L1 stimulation of the cells.

Our findings on the roles of STAT3 and STAT1 in mediating PD-L1 activities in breast cancer indicate that STAT3 and STAT1 cannot act independently from each other, and suggest that intersections exist between these two transcription factors in mediating PD-L1-induced effects. Indeed, concomitant roles for STAT3 and STAT1 were found in MCF-7 and MDA cells, and STAT3-STAT1 dimers were identified in the latter [17]. The binding of phosphorylated Y-705-STAT3 to PD-L1 was also observed under hypoxic conditions in MDA cells [35].

Moreover, our findings on STAT3 and STAT1 activation add one more layer to previous observations connecting these two transcription factors to other aspects of the PD-L1/PD-1 axis. The powerful activities of interferon γ, as the prime inducer of PD-L1 expression, were found to be mediated by STAT3, and in addition, STAT1 activation was found to up-regulate the expression of PD-L1 by tumor cells [16,17,18,19]. Furthermore, pSTAT3 gene signatures were correlated with PD-L1 expression in tumor cells and immune cells of breast cancer patient tumors, and the activated forms of STAT3 and STAT1 were significantly associated with the expression of PD-L1 in breast cancer patients [18,19].

As noted above, we have also provided the first evidence for the cardinal roles played by N-linked glycosylation at all four PD-L1 glycosylation sites in mediating the cell-autonomous pro-metastatic functions of PD-L1 in tumor cells. First, our studies with kifunensine demonstrated that the activation of STAT3 and STAT1 was effectively down-regulated by the inhibitor, alongside with a reduced tumor cell pro-metastatic functions. The inhibitory effects of kifunensine on PD-L1 activities in the cancer cells suggest that the activity of α-mannosidase I, which is the main enzyme inhibited by kifunensine [29,30], is pivotal for PD-L1-induced effects in breast tumor cells; our data also imply, via the use of swainsonine, that N-linked glycosylation by α-mannosidase II [29,30] plays a role in this process. This possibility was supported by the TCGA patient dataset findings, demonstrating the associations between high α-mannosidase II (MAN2A1) expression levels and reduced survival in breast cancer patients.

Then, analysis of the STAT3 and STAT1 activation patterns in PD-L1 that had been mutated at each of the four glycosylation sites individually demonstrated a complex connection between N-linked glycosylation of the different PD-L1 sites and their ability to induce pro-metastatic activities through STAT3 and STAT1 activation. Our findings revealed that in both MCF-7 and MDA cells, the N219 residue was the most crucial site for inducing STAT3 and STAT1 activation. Although less prominent, N-linked glycosylation of the N192 and N200 sites (depending on the cell type), but not of N35, also regulated STAT3 and STAT1 activation. Therefore, the N35 site probably regulates other signaling pathway/s or transcription factor/s that mediate/s the pro-tumoral activities of PD-L1 in the cancer cells.

Our findings on PD-L1 variants that had been mutated in each of the four N-linked glycosylation sites of PD-L1 indicate that all sites are required for PD-L1 pro-metastatic activities, and that the lack of glycosylation of one of the sites cannot be compensated for by any of the other three glycosylation sites. Moreover, our observations in the animal model system demonstrated that single alteration of even one of the four PD-L1 N-glycosylated sites—exemplified by the N35 and N219 sites—was sufficient to reduce the cell-autonomous T cell-independent pro-tumorigenic functions of PD-L1, also in vivo. These observations add significantly to a previous study using a PD-L1 variant mutated at **all four** N-linked glycosylation sites **concomitantly**, which demonstrated reduced growth of mouse TNBC tumors in a **T cell-dependent** system [21]. A mechanism connecting N-linked glycosylation of PD-L1 to cancer growth and anti-tumor T cell responses was also described in a syngeneic model system of EGF-induced responses [22]. Overall, our findings reveal that in addition to previous reports on the roles attributed to N-linked glycosylation of PD-L1 in regulating anti-tumor T cell activities [21], this post-translational process is required also for PD-L1 to act directly on the tumor cells in a cell-autonomous manner, leading to their increased aggressiveness.

Overall, our study indicates that N-linked glycosylation of PD-L1 is required for the ability of PD-L1 to induce cell-autonomous pro-metastatic activities in breast cancer cells, mainly by regulating the activation of STAT3 and STAT1.

## 5. Conclusions

In this study, we have identified key roles for members of the STAT family, namely STAT3 and STAT1, in mediating PD-L1-induced cell-autonomous and pro-metastatic activities in breast cancer cells. The roles of STAT3 and STAT1 in this system agree well with the potential use of STAT proteins as targets in cancer therapy, as has been suggested and addressed in different studies and in clinical trials [20]. However, the multifaceted roles of these two proteins in different cancer studies, and the fact that inhibition of STAT3 or STAT1 may indirectly affect many other processes, suggest that STAT3 and STAT1 inhibitors may give rise to unpredicted and undesired effects; additional limitations may also exist when STAT3 and STAT1 are targeted in clinical protocols combining ICBs [20,36,37]. Similarly, blocking enzymatic steps that are involved in N-linked glycosylation may lead to considerable adverse effects, because glycosylation is essential for the activity of many cellular proteins [38,39].

Instead, the fact that PD-L1-induced activation of STAT3 and STAT1 requires, as a prerequisite, that PD-L1 will be N-linked glycosylated, provides novel perspectives to PD-L1-directed therapy in breast cancer patients. Here, to follow up on observations showing that PD-L1 is N-linked glycosylated in breast cancer patient samples [23,24], it is important to validate the connections between the STAT3 and STAT1 proteins with the N-linked glycosylation status of PD-L1 in breast cancer patients and in mice tumor samples. The associations between these parameters and the survival rates of patients could also identify the degree of association between PD-L1 N-linked glycosylation and STAT3/STAT1 and therapeutic results.

Thus, further studies along these lines may lead to improved therapy approaches, directed to the glycosylated moiety of the protein. Recently, a 2018 paper by Hung and colleagues demonstrated that an antibody directed to the N192 and N200 glycosylation sites of PD-L1 (STM108) inhibited PD-L1 interactions with PD-1, and led to PD-L1 internalization and degradation; moreover, when the antibody was conjugated to an anti-mitotic drug, it reduced tumor growth in an animal model system using mouse breast cancer cells [21]. These findings agree well with our observations on the roles of N192 and N200 in regulating PD-L1 functions and STAT3/STAT1 activities in breast cancer cells, and offer new therapeutic opportunities that rely on targeted inhibition of the most active form of PD-L1, the one which is fully N-linked glycosylated.

Introducing antibodies to N-linked glycosylated PD-L1 in clinical use will require assessment of PD-L1 glycosylation levels in each patient biopsy beforehand. Such an analysis may serve not only therapeutic needs, but may also assist in stratifying patients for ICB treatment based on PD-L1 N-linked glycosylation levels. Recent reports indicate that the N-linked glycosylated form of PD-L1 is not well recognized by antibodies during diagnosis or by treatment with ICBs directed at PD-L1 [23,24,25,26], suggesting that such patients may have relatively lower responses to the therapy. In practice, it is possible that some of these patients do express PD-L1, and that the antibody binding site/s are hindered due to N-linked glycosylation. Such patients could benefit from diagnostic measures and therapies using antibodies to the glycosylated moieties rather than antibodies directed at the core protein itself.

## Data Availability

Not applicable.

## Supplementary Materials

The following supporting information can be downloaded at: https://www.mdpi.com/article/10.3390/cells12192338/s1, Figure S1: The cellular research systems used in the study; Figure S2: Expression characteristics of the 4A-PD-L1 variant in breast cancer cells; Figure S3: Associations of mannose-trimming enzyme levels with progression-free interval in breast cancer patients.
