# Supplementary material for: The Cell-Autonomous Pro-Metastatic Activities of PD-L1 in Breast Cancer Are Regulated by N-Linked Glycosylation-Dependent Activation of STAT3 and STAT1
Source: Cells. 2023 Sep 23;12(19):2338. doi: 10.3390/cells12192338 (PMC10571791; doi:10.3390/cells12192338)
Supplement: Supplementary file 1 [file cells-12-02338-s001.zip › cells-2586464-supplementary.pdf]

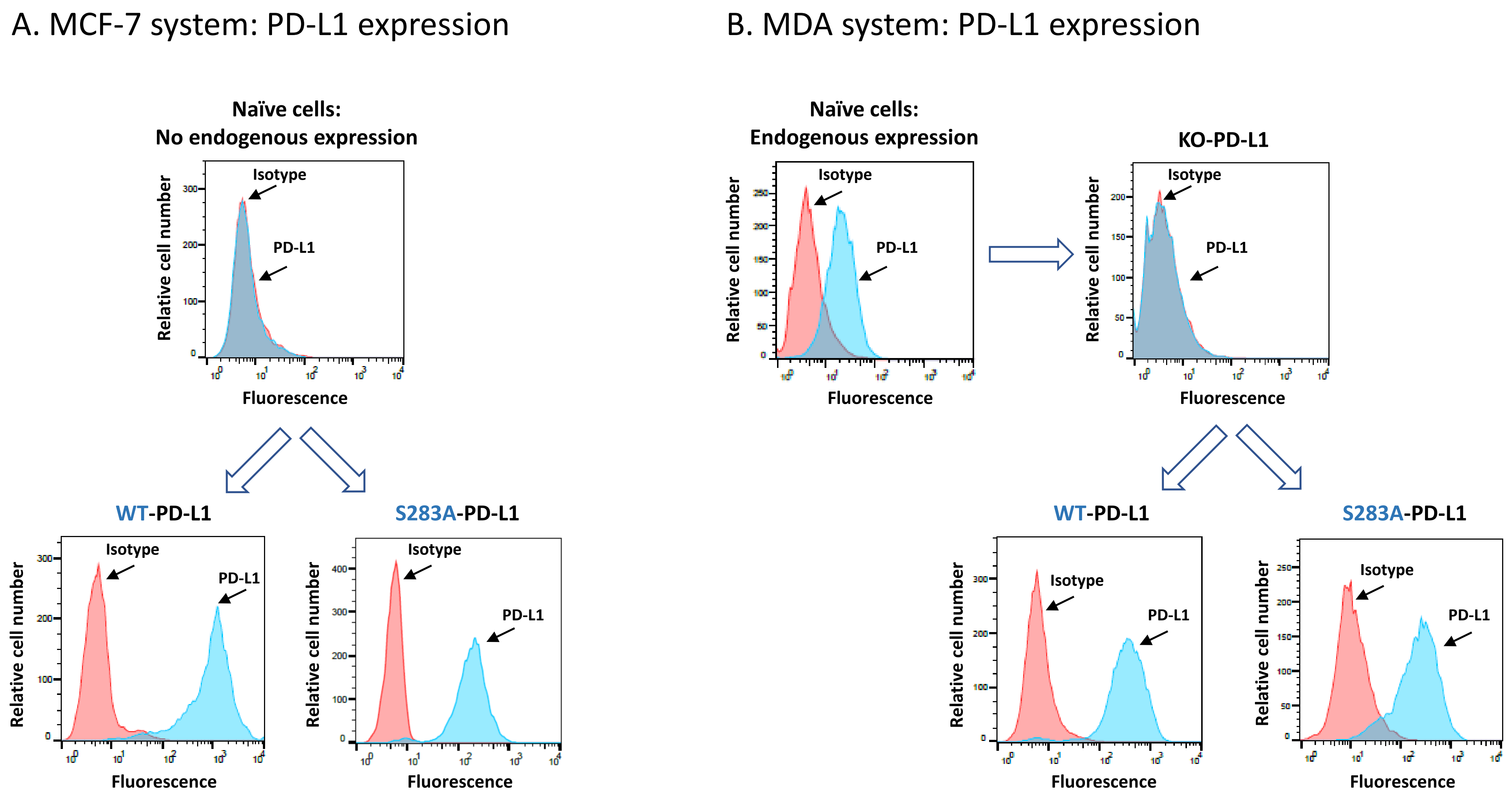

Figure S1

The cellular research systems used in the study

(A) Generating the MCF-7 cell system, composed of WT-PD-L1-MCF-7 cells and S283A-PD-L1-MCF-7 cells. MCF-7 cells, not expressing PD-L1 constitutively, were infected to express WT-PD-L1 or S283A-PD-L1 or vector only (PD-L1 expression in Vector control cells is demonstrated in different figures in the paper). The cells were then analyzed by flow cytometry for cell surface expression of PD-L1. Non-relevant isotype-matched antibodies were used as an isotype control. **(B)** Generating the MDA cell system, composed of WT-PD-L1-MDA and S283A-PD-L1-MDA cells. MDA cells express endogenous PD-L1. Thus, PD-L1 in these cells was first knocked-out (KO) by the Alt-R CRISPR-Cas9 method; PD-L1 KO was validated by flow cytometry of PD-L1 cell surface expression, and by out-of-frame sequence of PD-L1. Then, the cells were infected to express WT-PD-L1, S283A-PD-L1 or vector only (PD-L1 expression in Vector control cells is demonstrated in different figures in the paper). PD-L1 cell surface expression was validated in the resulting cell types by flow cytometry analyses. Non-relevant isotype-matched antibodies were used as an isotype control. The generation of the MCF-7 and MDA cell systems was described in detail in our published manuscript [10], and are presented again in the current study for readers' convenience. A different experimental repeat is presented in the current study, than in the previous publication [10].

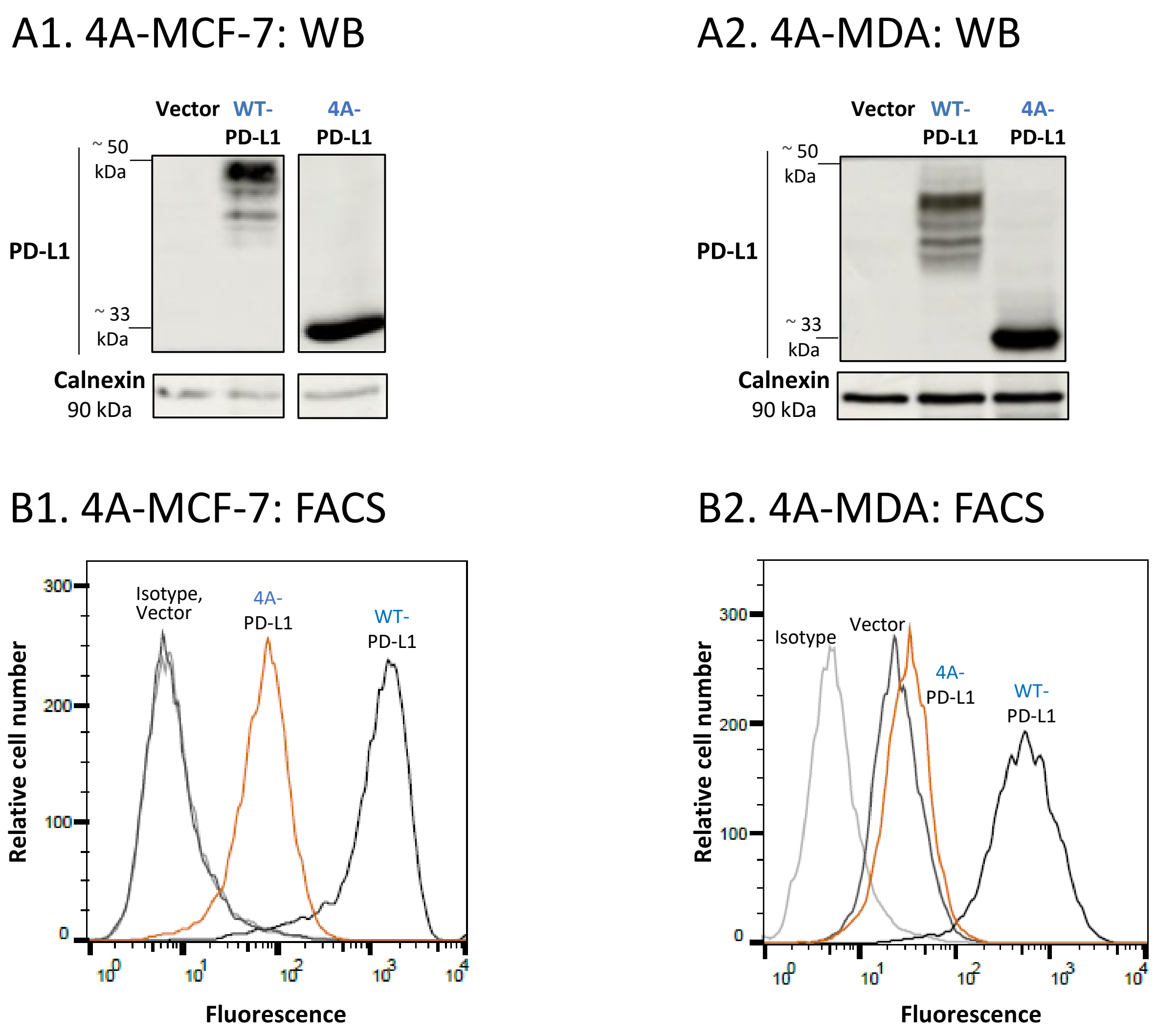

Figure S2

Expression characteristics of the 4A-PD-L1 variant in breast cancer cells

In parallel to MCF-7 cells and MDA cells expressing PD-L1 variants that were mutated in a single N-linked glycosylation site (N35, N192, N200, N219; Figs. 7 and 8), a variant mutated in all four N-linked glycosylation sites was produced, resulting in 4A-PD-L1-MCF-7 cells and 4A-PD-L1-MDA cells. **(A)** The Figure demonstrates PD-L1 expression in 4A-PD-L1 cells, compared to WT-PD-L1 cells and to control Vector cells, in MCF-7 cells **(A1)** and MDA cells **(A2)**, determined by WB. **(B)** The Figure demonstrates PD-L1 cell surface expression in 4A-PD-L1 cells, compared to WT-PD-L1 and control Vector cells, in MCF-7 cells **(B1)** and MDA cells **(B2)**. PD-L1 expression was determined by flow cytometry (FACS), using non-relevant isotype-matched antibodies as an isotype control. In all panels, the results are from a representative experiment of n=3, showing similar results.

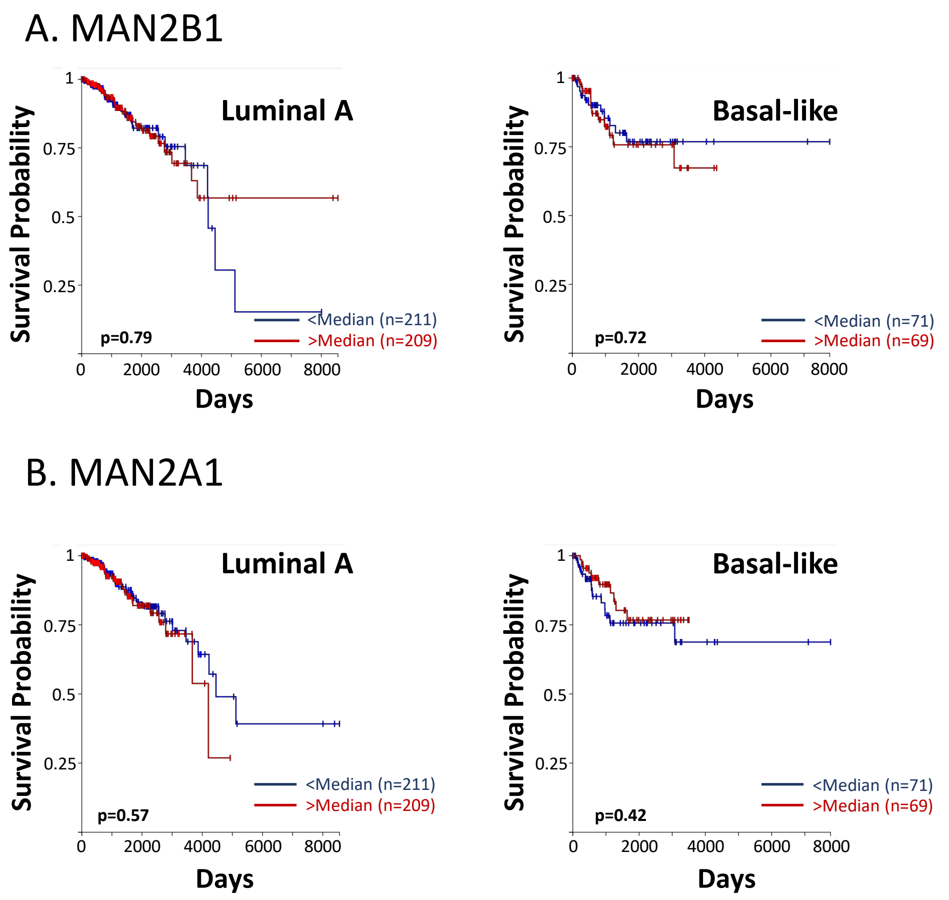

Figure S3

Associations of mannose-trimming enzyme levels with progression-free interval in breast cancer patients

The TCGA dataset was used to determine the connection between patient progression-free intervals and the expression levels of **(A)** α-mannosidase I (MAN2B1) and **(B)** α-mannosidase II (MAN2A1). In each patient group, "Luminal A" and "Basal-like", the patient cohort was divided based on median values of gene expression (above median = ">median"; below median = "<median"). Patient numbers in each group and p values are indicated in the graphs.
